# A Practical Compendium of the Diseases of the Skin, with Cases

**Published:** 1835-07-01

**Authors:** 


*A Practical Compendium of the Diseases of the Skin, with Cases.* By Jonathan Green, m.d. London, 1835. 8vo. pp. 371.

Books on the Practice of Physic admit of an obvious division into those which discuss the phenomena of some disease, or class of diseases, and those which investigate and define the powers of some remarkable remedy. The work now before us belongs rather to the second than to the first class; for, though it is not only judicious and instructive in substance, but lucid, and even elegant in style, its most striking merit consists in its exposition of the unrivalled efficacy of warm-bathing in cutaneous diseases. We would not, however, be understood to imply that the nosological part of the work is defective or erroneous; far from it. The reader, indeed, who merely recollects Dr. Green as the author of a very small tract on the advantages of bathing, will scarcely recognise his old acquaintance: the style, the tone of his precepts, the manner of relating the cases, are no longer the same; he will be gratified with this agreeable metamorphosis—lateritium invenit, marmoreum reliquit.

The sulphur-fume bath appears to be Dr. Green's favourite; and we have only to regret that no philanthropist has yet made this excellent remedy accessible to the bulk of the community.

Dr. Green, in his classification, has chiefly followed Willan and Bateman; but has adopted many of the improvements of Rayer and Biett, and has added several of his own.

We shall now proceed to touch upon a few scattered points in this Practical Compendium; which, from its nature, does not require a regular analysis. Our author commences his introduction with the following sentences. "It is my purpose in the following pages to present a systematic view of the diseases usually regarded as belonging more peculiarly to the skin. It is not my intention, however, to enter into a particular detail of the symptoms, treatment, &c. of every one of the species composing this class,—*many of them are held as falling within the province of the physician or surgeon generally, and are to be found treated of at length in every good system of medicine or of surgery*, and many of them are so rare, that, in this country, at least, their names are all that are known to us." (P. 1.) We confess that we do not fully comprehend the lines that we have marked in Italics; the exclusion is too sweeping, and Dr. Green puts himself out of court altogether, as the lawyers would say: if he will not enter into minute details concerning diseases which fall "within the province of the physician or surgeon generally," what is left for him?

The following are Dr. Green's accounts of the ordinary English practice in cutaneous diseases.

"All that has been done in regard to the treatment of this class of complaints in this country of late years amounts to a few experimental trials of certain heroic remedies, among which, mercury, arsenic, and prussic acid, figure in the foremost rank; and endeavours to force ourselves into the belief that cutaneous diseases were uniformly owing to some mysterious and indefinable affection of the digestive organs, nowise observable in nine cases out of ten, in any derangement of their functions, but for which the blue or Plummer's pill and purgative medicines were the approved specifics. One or other of these pills was therefore almost uniformly commenced forthwith, and ample doses of purgative medicine were prescribed. This course being persevered in for some time, and no good resulting, as was most frequently, though perhaps by no means invariably, the case, small doses of the hydrargyri oxymurias, combined with decoction of sarsaparilla, followed. This failing, in like manner, Fowler's arsenical solution, and perhaps decoction of dulcamara, were next recommended, and these either proving ineffectual against the disease, or causing some suspicious and unpleasant disorder of the system, were in their turn abandoned. The patience of the physician as well as of the patient (still truly patient in one, but no longer so in each sense of the word) being now worn out, they usually parted company at last, little satisfied with each other, the one lamenting the obstinacy of skin complaints, the other inveighing against the inefficacy of medicine, if not against the ignorance of its practitioners." (P. 2.)

"The treatment pursued in cutaneous diseases, the true end and aim of all preliminary study of their characters, has necessarily partaken of the obscurity that has hitherto prevailed, and that still prevails in regard to their nature. I have already hinted at the fact that a similar routine is indiscriminately followed in almost every one of these affections; in France a course of bitters and sulphur, in England one of mercury (in one or other of the infinite variety of forms into which it has been tortured by ingenuity) and of purgatives, are the approved and universally received specifics; it matters not that disappointment again and again attends the prescription of these medicines, the mind once familiarized with a favourite notion is not often emancipated from its empire even with the evidence of its falsity; and then the diseases of the skin were held such rebellious affections, that the medicines were generally excused for their want of efficacy, and prescribed as before in the next case that occurred." (P. 13.)

It is unnecessary to point out the inconsistency of the two accounts; but even the more ample and liberal one gives a very imperfect list of the remedies employed by English physicians in skin diseases. Nothing is said in it of the use of

Liquor Potassæ in Psoriasis, of Dr. Beck's treatment of Lepra by tar administered internally; of the cure of syphilitic eruptions by iodine; or of the use of external stimulants in porrigo. The effects of sulphureous baths, too, in lepra and psoriasis, have been tried by Dr. Bardsley on a very large scale. It is quite clear, from a hundred passages in this book, that Dr. Green is well acquainted with almost every point of British practice relating to cutaneous diseases; and he is, therefore, the less excusable for putting together the hasty and erroneous burlesque of it which we have just quoted.

In treating of "the effects of therapeutic agents," Dr. Green, of course, dilates at great length upon the advantages of bathing; far from finding fault with this, we are extremely pleased with it; partly because nothing can be better than the minuteness, and even the prolixity of enthusiasm on a subject where the writer is qualified to appreciate, not only the broader colours, but the more delicate shades; and partly because this branch of therapeutics is inadequately valued by the mass of the profession, as well as of the public. Holding these opinions, therefore, we make no apology for the length of the following quotation.

"The simple cold or tepid bath frequently gives great relief in many diseases of the skin, greatly allaying the itching, and state of nervous irritation that attend them, and thus conducing to the ultimate cure. I am inclined to believe that the value of sea-bathing is greatly overrated in its effects upon affections of the skin. I have seldom known it accomplish any permanent good in this class of complaints, and, on the contrary, I have often heard the origin of different forms of skin disease ascribed to its influence. Baths of the natural mineral waters have been long known to prove very serviceable in many diseases of the skin. These are susceptible of being closely imitated by art; we can, indeed, in this way produce more powerful and more speedy effects than result from bathing in the natural mineral springs. The artificial sulphureous water bath, with a quantity of gelatine or fine glue dissolved in it, is one of the best baths known in many inveterate diseases of the skin.

"But every form of water bathing that has been tried falls immeasurably short of the hot air and vapour bath, in its immediate and powerful curative influence on the great majority of the diseases of the skin. The hot air and vapour bath may very properly be spoken of together, inasmuch as their effects on the system are very nearly similar. I am in the habit of administering the hot air bath to patients at first, at the temperature of about 98° of Fahrenheit, and of raising it gradually in the course of from fifteen to twenty minutes to 110°, and, if the full effect of the bath is not obtained, to 120°, or even 130°, of the same scale. The patient seated in the apparatus and exposed to this degree of heat, is only sensible at first of a slightly increased but pleasant warmth. Within a few minutes the expression becomes cheerful and animated, the eyes sparkle, the countenance looks florid and then flushed, the pulse rises in frequency, and gains much in fulness, but is soft; the whole body, the face (which of course is not enclosed) as well as the other parts, next become bathed in perspiration, so that the sweat is seen standing in beads upon the forehead and trickling down the cheeks. The patient is now no longer sensible of any increase of temperature, although he is perhaps exposed to a heat of 150 degrees of Fahrenheit. It is matter of astonishment to the generality of persons how such a temperature can be borne without injury. But it is in virtue of the same physical law which fits man to become the denizen of lands within the tropics and of regions near the pole. All increase of activity in the vital processes of respiration and circulation, the furnace and flue of the system, induced by stimulus of any kind, especially by that of augmented temperature, is accompanied with a commensurate increase in the exhaling functions of the skin, and of consequent evaporation from its surface, a process the cooling effects of which are familiar: the strata of hot air immediately in contact with the body are successively robbed of their excess of heat, by the conversion of the watery products of perspiration into vapour, in which the caloric that was sensible, and that tended to raise the temperature of the body, immediately becomes latent. There is consequently no means of cooling a hot air bath, say of 120 degrees, down to its own temperature 98 degrees, so effectually as the immersion within it of a living human body; and this is the reason that the temperature falls so rapidly in ill-constructed baths, and that it has to be kept up so incessantly even in those of the best construction.

"After the perspiration has appeared about five or six minutes on the forehead, the full effect of the bath has been obtained, and the patient should immediately quit the apparatus. If the stimulus be continued longer it is at the expense of the agreeable feelings first induced; a degree of languor and exhaustion succeeds to these, and patients then feel drowsy and disposed to sleep. But if the bath be quitted when the effects are at their height, a comfortable degree of warmth is experienced for some hours afterwards, and the activity of the body and the elasticity of the mind, far from being diminished, are on the contrary very much increased. It sometimes happens that the skin is in so dry and unperspirable a state, that a moderate degree of heat in the bath fails to induce sensible perspiration, and then, if the temperature be allowed to rise rapidly, patients complain of an unpleasant scorching sensation. In these cases, a little watery vapour let into the bath by an apparatus contrived for the purpose, gives immediate relief, and very speedily induces the state of surface we are desirous of obtaining." (P. 18.)

Perhaps, if others have overrated the value of sea-bathing in cutaneous diseases, our author places it too low: we would rather incline to the golden mean of Hufeland, who thinks it commendable, provided that it is preceded by the use of internal remedies, that the disease is merely local, and that the course is began with warm sea baths and the temperature gradually diminished. (Endlich verdient es auch bey *chronischen Hautkrankheiten* empfohlen zu werden; doch mit der Vorsicht, dass vorher ein gehöriger Gebrauch innerlicher Mittel gemacht, und die Krankheit nur noch blosse Localkrankheit sey, und auch dann, dass man erst mit erwärmten Seebädern anfange, und allmählig zum kalten übergehe.—*Hufeland. Praktische Uebersicht der vorzüglichsten Quellen Teutschlands. Das Seebad.* S. 266.)

After cautioning us against employing the hot-air and other stimulating baths in the acute stages of several cutaneous diseases, such as eczema, ecthyma, impetigo, &c. he remarks that they are excellent remedies in cases where there is a shattered constitution without any tangible disease. The warm bath, in fact, is the best palliative for that incurable malady, old age, and is recommended, as we recollect, in the very strongest terms by Gregory in his Conspectus Medicine theoreticæ. Why did not Darwin, who saw that Prometheus was the first distiller, and that the vulture devouring his liver shadowed forth the hepatic diseases of dram-drinkers, discover also that Medea's cauldron was a sulphureous bath at 100°?

"Dr. R., aged eighty-five, for nearly thirty years senior physician to the largest hospital in this country, had long been in very indifferent health, and affected with a constant nervous shaking of the arms, when he was incidentally persuaded to try the effects of the sulphur fume bath for a troublesome impetiginous disease of the legs. Under the use of this remedy, not only did the affection of the legs gradually disappear, but such a signal improvement took place in the general health, that the doctor declared he thought if he had known and made use of the sulphur fumigations sooner, he might very possibly have extended his life to a hundred years. In little more than a month, he told me that though for years he had scarcely been able to digest any thing, not even a potato, unless boiled until ready to fall to pieces, he now thought he could eat a raw carrot without inconvenience." (P. 22.)

Having passed through the introduction, we now arrive at the body of the work; the first division of which treats of the inflammatory affections of the skin; and the first subdivision of this, again, is appropriated to the exanthemata.

The Diseases placed under the head of *Exanthemata* are Erythema, Erysipelas, Roseola, Rubeola, Scarlatina, and Urticaria.

We find two cases of Erythema treated by the vapour bath.

"A young lady of fair complexion, of a sanguine but very nervous temperament, after considerable exertion, found herself covered on the upper parts of the body and neck, as well as over the arms, with red patches, varying in form and size, and showing in their centres whitish and hard elevations, similar to gnat-bites. In some places the patches from running together produced a curious marbled appearance of the surface. The complaint was attended with much inconvenience but little pain, and had existed more than a month when I saw the patient for the first time. There was a regular aggravation of the symptoms every day; the small whitish tumours then became larger, and the redness of the other parts assumed a darker hue; the surfaces affected then looked shining, tense, dry, and as if swelled; the red colour fading towards the healthy parts. The patient had little appetite, and her nights were sleepless, or her rest was broken; the tongue was covered slightly with greyish mucus, and she complained of extreme lassitude. I advised a trial to be made of the simple vapour bath; medicine did not seem to be needed. After the third bath, the patient fell into a profuse perspiration; the skin then became soft and moist to the touch. The parts which had been the seat of the disease became wrinkled and faded, the small elevations gradually shrunk away, and in ten days there were no traces of the disease left.

"A gentleman of a robust and sanguine temperament, aged about fifty, received a hurt in his back by a fall from his horse, for which it was treated as usual in such cases. Amongst the means resorted to, he had used the essence of mustard (spirits of turpentine) as an embrocation, and to this was attributed a very general attack of erythema, extending from the forehead to the ancles, with which he was immediately afterwards seized; but the back and parts rubbed by the embrocation, and fretted perhaps by the close contact of the clothes, were those which were more particularly affected with this superficial inflammatory blush. Even in the midst of these, however, there were some linear spaces where the skin appeared almost as in health; neither, indeed, did the very reddest parts of the skin look tense or swollen, but simply as if they had been stained with a red, and in some parts purplish red, ink. The constitutional derangement was not severe; but there was still considerable depression of strength and spirits, loss of appetite, small and frequent pulse, tongue covered slightly with mucus at the base. The inflamed surface was uniformly extremely tender, and felt, as the patient expressed himself, as though hot water had been applied to it. Gentle laxatives and diaphoretics, with the use of the vapour bath daily, proved sufficient to remove all the symptoms within the fortnight; the patient then, for a short time, took the nitric acid in small doses, as a tonic, and soon became well." (P. 31.)

Our author even ventures to recommend the sulphur fumebath in erysipelas, after the first violence of the symptoms has been mitigated by the antiphlogistic system. He gives a case, in which he adopted this bold practice with great success. The patient was a lady, aged forty-five, of a weak and flabby constitution, who for some time had suffered two or three attacks of erysipelas every year. On the 12th of October, 1831, Dr. Green saw her for the first time; and, on the 15th, the symptoms having diminished in severity, she took the bath for the first time. It produced immediate relief, was repeated with great benefit, and would seem to have strengthened the vital powers permanently, as the disease has not returned.

Under the head of Scarlatina, our author recommends the vapour bath as the best means of arresting the anasarca by which this disease is sometimes followed. He adds his testimony to those which have so often been published lately, of the prophylactic powers of belladonna; but the sentence in which his evidence is couched is ambiguous: "I had very lately an opportunity of witnessing the good effects of this medicine among the children assembled at a boarding-school, where scarlatina broke out; four of the children to whom the medicine was administered escaped the disease entirely." (P. 61.)

Was the medicine administered to four only; or, out of a greater number to whom it was given, did four escape the disease?

The next division (or order) is *Vesiculæ*, which includes Scabies, Herpes, Eczema, and Miliaria. Dr. Green includes Scabies in this division (though placed by Bateman among pustular diseases,) because it is now pretty generally admitted to be vesicular in its primary form; and he excludes Vaccinia and Varicella, from their obvious affinity to the varioloid group.

*Bullæ* form the third division, containing two genera, Pemphigus and Rupia.

*Pustulæ* form the fourth division, containing seven genera, Variola (including Varicella), Vaccinia, Acne, Mentagra, Ecthyma, Impetigo, and Porrigo.

The following cases of impetigo are interesting and instructive.

"Case ii. A gentleman, nearly sixty, of a gross, robust habit, applied to me, in the summer of 1832, on account of an eruption on the skin, from which he had suffered more or less during the last eight years. The eruption consisted of numerous small yellow pustules on the insteps, ankles, and lower parts of the legs, which were now much swollen, and encased with lamellar crusts, impetigo *sparsa*: the hands and several parts of the trunk of the body were similarly affected. The patient's chief complaint arose from the itching, which was at times very annoying; otherwise he was in fair health, and went about his affairs, though it often required the exercise of considerable ingenuity to apply a sufficiency of rag in such a manner as to absorb the abundant sero-purulent discharge that issued from the parts affected with the disease. June 12th, the patient was bled to the amount of sixteen ounces, and was directed to take every morning four table spoonfuls of an aperient mixture consisting of infus. sennæ, ℥vi., tinct. ejusdem, ℥i., syr. rhamni ℥i. His diet was also regulated, and a sulphur fume bath administered daily.

"The gross habit of this patient seemed to require bleeding and purging; otherwise the shining redness and heat of skin that attend impetigo should not deter from the use of sulphur fumigation, which, although apparently counterindicated, I never knew otherwise than of the greatest use in this complaint: let the external appearances of inflammation be never so great, it is surprising how soon the discharge and redness begin to abate under the use of this application.

"On the 30th, not a vestige of the disease was to be seen, except between the thumb and fore finger of each hand, where it had probably only been kept up, by the patient picking the parts with his nails; all itching had ceased, and for the week previous the opening mixture had been discontinued. The fume baths were also soon discontinued, as no longer needful; the patient appeared restored to perfect health.

"To celebrate this redemption from his long continued and harassing complaint, my worthy patient gave a grand feast to his numerous friends, who were invited to rejoice with him on occasion of his happy recovery. Moderation is difficult in the moment of victory, and my patient committed an excess, which after twenty-four hours brought on an almost general attack of inflammation of the skin, followed by a copious eruption of pustules; so that he returned, depressed in spirits, to resume the former treatment. In the short space of eight days this active and general accession of disease was completely subdued, and all remedies were left off.

"I have had an opportunity of frequently hearing of this patient: he has had no relapse of his disease worth naming; a few pustules on the hands are all he has seen of it since he was under my care. I have been rather surprised at this, for I know that his habits are such as are most commonly held to be inimical to complete recovery from chronic affections of the skin especially.

"Case iii. Mrs. H. of Faversham, aged seventy-four, was recommended to try the effects of sulphureous fumigation under my direction, by the medical gentlemen she had consulted in London, Dr. Gordon and Sir Benj. C. Brodie, on account of an eruption of impetiginous pustules, extending over the whole surface of the body, except the face, the palms of the hands, and soles of the feet, under which she had laboured during the last four years. From the nape of the neck and throat to the heels and insteps, the surface of this gentlewoman's body was generally inflamed and covered with small yellow-headed pustules and laminated incrustations, from under which a profusion of sero-purulent fluid was constantly pouring. The itching and general irritation of the surface were very distressing, so that the patient scarcely ever enjoyed an hour's quiet and uninterrupted sleep.

"In spite of the apparently high state of inflammation, the sulphur fumigating bath was immediately entered; and with so happy an effect, that the succeeding night was tranquil, and a considerable portion of it passed in refreshing sleep. After the fourth bath the amendment was so great, that the patient, with a view of accelerating the cure still more, insisted upon taking two baths daily. In the short period of a fortnight, during which time Mrs. H. had taken twenty-two baths, not a vestige of this extensive disease was to be seen, and she returned into the country quite recovered." (P. 148.)

The fifth division consists of *Papulæ*, of which the genera are Lichen, Strophulus, and Prurigo. In this last disease the sulphur fume-baths are of extraordinary advantage, but it is often requisite to premise bleeding and purging; otherwise what ought to be the remedy becomes the aggravation of the disease.

*Squamæ.* This order includes the genera Lepra, Psoriasis, and Pityriasis. In treating of lepra, our author says:

"If the disease be of recent date and attended with considerable inflammation, itching, and constant uneasy sensations in the patches, general bloodletting and emollient measures of various kinds, such as the tepid gelatinous or gruel bath, the simple vapour bath, and the application of cream or hog's lard to the irritable surfaces, will be found to give great relief, and of themselves frequently dispose the disease to recovery. * * *

"Simply irritating applications, such as pitch plasters, tar ointment, salves of the bryonia alba, chelidonium, &c. which used to be mostly employed in cases of leprosy by our forefathers, are now acknowledged to be even worse than useless, often positively aggravating the disease." (P. 204.)

We certainly would not recommend stimulants, if the inflammation were *considerable;* but, in ordinary cases, our own experience agrees with the axiom of Heberden, that itching eruptions are to be treated with stimulating applications: and our author, though he disapproves of tar-ointment, yet immediately afterwards recommends such remedies as ointments of nitrate of mercury and ioduret of sulphur.

Dr. Green has only had three incurable cases of lepra:

"One was that of a gentleman who took the sulphur fume bath upwards of a hundred times, without deriving much or any permanent good from it. Another gentleman, after a trial to the same extent of this means, and a similar result, went to India, and I heard no more of him. The third is a medical gentleman, who has had the disease from puberty, and is now fifty years of age. He too has taken more than a hundred fumigations, though very irregularly, and considers himself incurable of the disease in question; otherwise he is in fair health." (P. 213.)

*Tubercula,* the seventh order, contains five principal genera; they are, Lupus, Greek Elephantiasis, Cancer, Molluscum, and Frambœsia.

*Furunculi,* the eighth order, contains Furunculus, Anthrax, and Pustula maligna.

After the discussion of the eight regular orders, we come to those cutaneous diseases which appear under a variety of elementary forms; and, as our author has very properly excluded Burns and Chilblains from this division (into which they are thrust by Rayer and Alibert,) it contains only syphilitic eruptions. We shall content ourselves with extracting a single case:

"Captain A. B., of the Guards, placed himself under the care of Mr. Earle, when already reduced to extremities by the united influence of secondary syphilis, and the constitutional disturbance induced by the ill-timed use of mercury. The forehead and limbs were covered with numerous foul superficial sores, and the parietes of the abdomen and thorax with ulcers of such depth, that several of them seemed to penetrate to the peritoneum and pleura. One side of the scrotum and one testicle had sloughed away, before I saw the patient; the other was lying naked, hanging by the cord, when he first visited me. There was also extensive superficial ulceration of the throat, and the shins were occupied with several nodes. The patient complained of severe suffering from nocturnal pains, and was greatly reduced in strength and spirits, and much emaciated, although in the prime of life.

"All that unwearied attention and the best advice could do in this case were done, but no decided improvement took place, and the state of the general health was such, that Mr. Earle felt it impossible again to have recourse to mercury. As a last measure, therefore, and with a view of arousing the drooping powers, he recommended a trial to be made of the sulphur fume bath.

"The first three exposures in the fumigating apparatus occasioned a good deal of smarting of the open sores, and seemed even to increase the restlessness and general distress endured in the night: on this account opiates were prescribed, and with the best effects. After the fourth fume bath had been taken an evident amendment was visible. The patient felt stronger, and generally better; his appetite began to return; several of the smaller sores had healed, and others were in progress of cicatrization; his spirits also rose, and his hopes of ultimate recovery revived. The baths were left off after the fifteenth, for ten days, in consequence of an attack of diarrhœa, which, however, did not interfere with the patient's improvement; for, on his return, almost the whole even of the very deepest ulcers had cicatrized. The remaining testis was nearly surrounded with a new integument; and Captain A. B. was gaining flesh and strength so rapidly, that he very speedily declared himself quite recovered. As a measure of precaution, small doses of the hydrarg. muriat. were continued for about six weeks afterwards; and as several years have now elapsed without any return of syphilitic symptoms, it seems probable that the poison was completely eradicated from the system.

"The remaining testicle was uninjured: Captain A. B. is married since his illness, and the father of four healthy children."

We shall pass over the remainder of Dr. Green's book, and conclude with a very large extract from his "useful formulæ."

BATHS.

*Sulphureous Water, or Artificial Barrèges Bath.*

℞. Sulphuret. potassæ, lbi.

Aquæ, Cong. xxx. M.

*Sulphureo-Gelatinous Bath.*

℞. Sulphuret. potassæ, ℥ij—℥iv.

Aquæ, Cong. xxx.

Add to this solution,

Ichthyocollæ, lbi—lbij.
in aquæ bullientis soluta lbx. M.

"This bath is preferable to the artificial Barrèges bath, as it is neither irritating, nor apt to occasion feverishness, which the common sulphureous water bath is.

"A cheaper and not less efficacious gelatine may be procured by dissolving from lbiss to lbii of parchment clippings in water, by long boiling, or by using a neat's or calf's feet for the purpose.

*Emollient Bath.*

"To an ordinary tepid water bath add a large basinful of thick gruel or paste, and mix it well with the water.

"One or other of these baths is often of great use in prurigo, eczema, lichen, and impetigo.

*Nitro-Muriatic Acid Bath.*

℞. Acid. nitrici, ℥iij.

Acid. muriatici, ℥i. M.

"To be added to the water of a tepid bath, which should then be about as sour as distilled vinegar.

*Sublimate Bath.*

℞. Hydrarg. chloruret (oxy-muriat.), ʒii—℥i.

Aquæ tepid. Cong. xxx.

"Sometimes prescribed in syphilitic affections, when we would avoid the action of the medicine on the stomach. Thirty are said in general to be sufficient for the cure. Formulaire de l'Hôpital des Vénériens.

*Alkaline Bath.*

℞. Potassæ sub-carbon. ℥iv—℥viii.

Aquæ tepid. Cong. xxx. M.

"Very useful in promoting desquamation from the skin, and in allaying pruritus in several forms of prurigo especially.

*Artificial Harrowgate Bath.*

℞. Sodæ muriat. lbij.

Magnes. sulph. ʒiij.

Potassæ sulphuret. lbi.

Aquæ, Cong. xxx.

FUME-BATHS, OR FUMIGATIONS.

*Sulphur.*

Sulphur. sublimat. ℥i.—℥ij—℥iij.

*Chlorine.*

℞. Oxid. nigr. manganes. ℥ss.

Acid. muriat. ℥i.

"It is sometimes advisable to substitute this bath occasionally for that of the sulphur fumes, when the disease of the skin, for which these are prescribed, proves very rebellious. It soon occasions the mouth to become sore, like the nitro-muriatic acid bath.

*Mercurial.*

Hydrarg. oxid. cinerei, ʒi—ʒij—ʒiij.

"The grey oxide is preferable to the red sulphuret of mercury or cinnabar, as it does not occasion coughing when inhaled, which cinnabar always does. It is, on the same account, greatly superior to cinnabar for fumigating ulcers in the throat.

*Aromatic.*

℞. Gum. benzoin. ʒiv—℥i.

"Aromatic effluvia may also be raised in the heated air bath from any other of the fragrant gum resin, essential oils, &c.

LINIMENTS AND LOTIONS.

℞. Potassæ sulphuret. ʒiij.

Sapon. mollis, ℥i.

Aq. calcis, ℥viij.

Spirit. vin. rect. ʒij. M.

"This is a good wash in porrigo especially, but is also useful in many other species of cutaneous disease.

℞. Liq. potassæ, ʒij.

Ol. oliv. ʒiij.

Aq. rosæ, ℥i. M.

℞. Liq. potassæ, ʒij—ʒiv.

Aq. rosæ, ℥ii. M.

"These are both of great service in cases of obstinate lepra and psoriasis especially.

℞. Acid. nitrici,

Acid. muriat. *aa* gtt. xx.

Aquæ rosæ, ℥vi. M.

"This may sometimes be used with good effect in cases of pityriasis and of chloasma.

℞. Hydriod. potassæ, ʒss.

Spirit. tenuior. ℥i.—℥iv.

Aq. rosæ, lbss. M.

℞. Hydrarg. chloruret. (corrosive sublimate,) gr. viii.

Aquæ rosæ, lbi.

Spirit. vini rectif. ℥i—℥ii.

"These are both excellent lotions in cases of acne. They may be made with emuls. amygd. amar. instead of rose water.

*Eczema of the fingers.*

℞. Hyd. oxy-muriat. gr. ij—vj.
Spirit. vin. rect. ℥i. M.

*Impetigo, Eczema, Lichen, &c.*

℞. Liq. plumb. sub-acet. ʒi.

Spirit. vin. rect. ℥i.

Aq. distill. lbi. M.

℞. Acid. hydrocyanici, ʒij.

Hyd. oxy-muriat. gr. iij.

Mist. Amygdalæ, ℥vj. A. T. THOMSON.

*Impetigo, &c. &c.*

℞. Sulphat. alumen.

———— zinci, *aa* ʒi.

Aq. ferventis, lbss. M.

Adde acid. sulphur. ʒss.

℞. Acid. hydrocyanici, ʒij.

Plumb. acetatis, gr. xvj.

Aq. distill. ℥viiss.

Spirit. vin. rect. ʒij. M. A. T. THOMSON.

℞. Hydrargyri, ʒi.

Acid. nitrici, ℥ii.

Aq. distill. lbv.

"Treat the mercury with the nitric acid, and complete the solution by adding the distilled water; half an ounce is used morning and evening as a lotion in scabies, prurigo formicans, &c. It does not stain the linen. The solution of the mercury in an excess of nitric acid is one of the best caustics and escharotics we possess, in arousing indolent sores generally, and in arresting the morbid actions of phagedenic ulcers, as of lupus, &c.

"A solution of uniform strength for use as a caustic, may be prepared as follows:

R. Hydrarg. proto-nitrat. sicc. ʒi.

Acidi nitrici, ℥i. M." (P. 352.)

Upon the whole, we are much pleased with this book: it cannot fail to be instructive, as it is replete with the results of long and successful practice.

